# Complete Genome Sequence of the Plantaricin-Sensitive Strain Lactobacillus plantarum NCIMB 700965

**DOI:** 10.1128/MRA.01724-18

**Published:** 2019-05-23

**Authors:** Dustin D. Heeney, Maria L. Marco

**Affiliations:** a Department of Food Science & Technology, University of California Davis, Davis, California, USA; University of Rochester School of Medicine and Dentistry

## Abstract

Lactobacillus plantarum NCIMB 700965 was isolated from cheese in 1939 and is used as an indicator strain for plantaricin production. The complete genome was determined using both long (PacBio) and short (Illumina) read data resulting in a single, circular chromosome with 3,015,426 bp, a G+C content of 45%, and five plasmids.

## ANNOUNCEMENT

Lactobacillus plantarum is known for its probiotic properties and necessity in the production of many fermented foods. Strains of this species synthesize a variety of bactericidal peptides called plantaricins that cause cell death in closely related bacteria (1, 2). Strain NCIMB 700965 (also known as NCDO 965, NCFB 965, and CECT 4645) was originally isolated from cheddar cheese in 1939 (3). This strain has improved our understanding of *L. plantarum* arginine metabolism and CO_2_ requirements (4). Because of its sensitivity to *L. plantarum*-produced bacteriocins, also called plantaricins, NCIMB 700965 has also been used as an indicator for plantaricin biosynthesis by other *L. plantarum* strains (1).

Strain NCIMB 700965 was acquired from the National Collection of Industrial Food and Marine Bacteria (NCIMB) and grown in lactobacilli MRS (BD Difco, Franklin Lakes, NJ) medium. Genomic DNA was isolated and sequenced as previously described (5). Briefly, a PacBio RS II instrument (Pacific Biosciences, Menlo Park, CA) with an insert size of 10 kb and P6C4 chemistry was used according to the manufacturer’s instructions. Initial *de novo* assembly was accomplished with a minimum seed read length of 6 kb, a minimum subread length of 1 kb, a genome size of 3.4 Mb, and target coverage of 15×. Contigs generated from Illumina short read data (SRA accession number SRR1553345) were aligned to the PacBio assembly data (RS_AHA_Scaffolding.1) to generate a single high-confidence FASTA file. Chromosome and plasmid circularity were confirmed with Gepard version 1.4 (https://github.com/univieCUBE/gepard/tree/master/dist). Overlaps were trimmed in SeqBuilder Pro 15 (DNASTAR, Madison, WI). The final assembly resulted in an average 310× coverage with one chromosome (3,015,426 bp; G+C content, 45%) and five plasmids, namely, plasmid 1 (66,439 bp; G+C content, 42%), plasmid 2 (52,109 bp; G+C content, 40%), plasmid 3 (41,818 bp; G+C content, 40%), plasmid 4 (23,484 bp; G+C content, 41%), and plasmid 5 (16,940 bp; G+C content, 38%). According to RAST annotations (6), the NCIMB 700965 genome contains 2,929 coding sequences and 68 tRNAs on the chromosome and 90 genes on plasmids. Genome alignments using BLAST showed that strain NCIMB 700965 is closely related to Lactobacillus plantarum SN35N (GenBank accession number AP018405; query cover, 93%; identity, 99%), a strain isolated from fresh pear (7).

Strain NCIMB 700965 is unable to synthesize functional plantaricins according to BAGEL4 (http://bagel4.molgenrug.nl/) (8). Compared with the well-characterized *L. plantarum* WCFS1 genome (9), it was noted that only *plnE* (encoding one component of the plantaricin EF bacteriocin) is intact in NCIMB 700965 out of 23 genes encoding plantaricin bacteriocins, immunity proteins, transporters, sensor kinases, and transcriptional regulators. Although NCIMB 700965 contains the plantaricin EF immunity gene *plnI*, an IS*30* transposon (10) is located near the 5′-terminal end, which results in a premature stop codon. The other genes in the plantaricin locus either contain mutations compared with WCFS1 (*plnF*, *plnG*, *plnH*, *plnU*, *plnV*, and *plnW*) or are absent from the genome (*plnA*, *plnB*, *plnC*, *plnD*, *plnJ*, *plnK*, *plnL*, *plnM*, *plnN*, *plnO*, *plnP*, *plnQ*, *plnR*, *plnS*, and *plnT*). These data confirm that NCIMB 700965 is a useful indicator strain for plantaricin production and can aid in the elucidation of how these bacteriocins kill sensitive cells.

### Data availability.

The complete genome sequence of NCIMB 700965 has been deposited in DDBJ/ENA/GenBank under GenBank accession numbers CP023490 (chromosome) and CP023491 to CP023495 (plasmids 1 to 5), BioProject accession number PRJNA407985, and SRA accession number SRX3207236.
